# Supplementary material for: Site-specific phosphorylation of TRANSPARENT TESTA GLABRA1 mediates carbon partitioning in Arabidopsis seeds
Source: Nat Commun. 2018 Feb 8;9:571. doi: 10.1038/s41467-018-03013-5 (PMC5805785; doi:10.1038/s41467-018-03013-5)
Supplement: Supplementary file 1 — Supplementary Information [file 41467_2018_3013_MOESM1_ESM.pdf]

Site-specific phosphorylation of TRANSPARENT TESTA GLABRA1 mediates carbon partitioning in *Arabidopsis* seeds

Li et al.

SUPPLEMENTARY INFORMATION

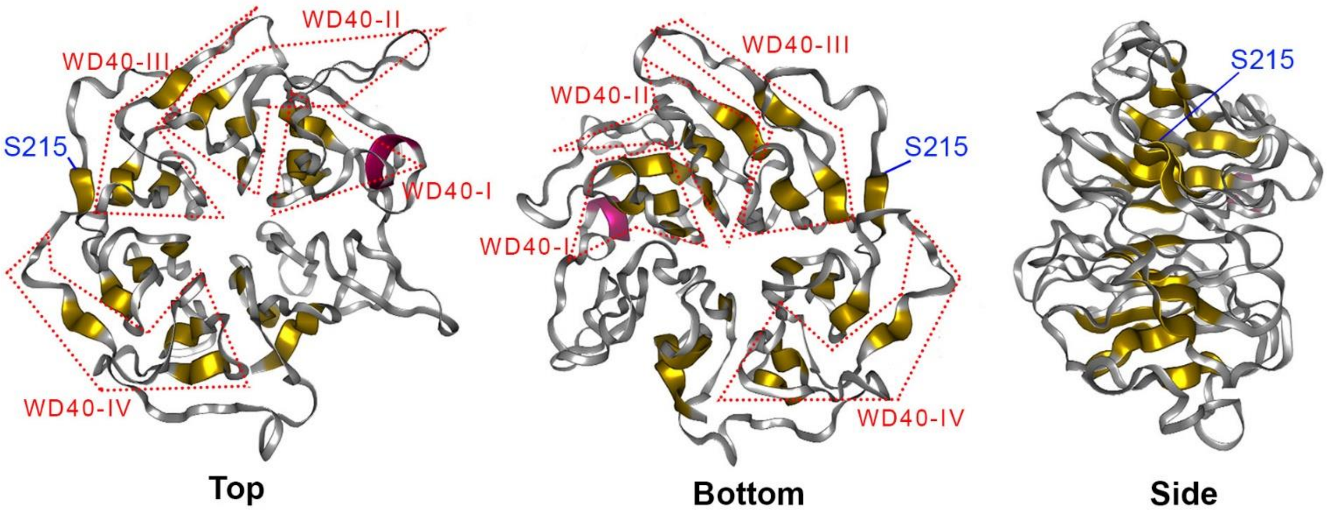

Supplementary Figure 1. Structure modeling of TTG1. Predication of TTG1 protein structure was performed with Protein Homology/analogY Recognition Engine V 2.0 (Phyre^2) available on the website (http://www.sbg.bio.ic.ac.uk/phyre2/html/page.cgi?id=index). Top, bottom, and side views of the TTG1 protein structure are shown. β-sheets and one α-helix are indicated in yellow and magenta, respectively. The four regions that approximately delineate four WD40 repeats in TTG1 are marked as WD40-I, WD40-II, WD40-III and WD40-IV, respectively. Serine 215, which is phosphorylated by SK11/12, is highlighted.

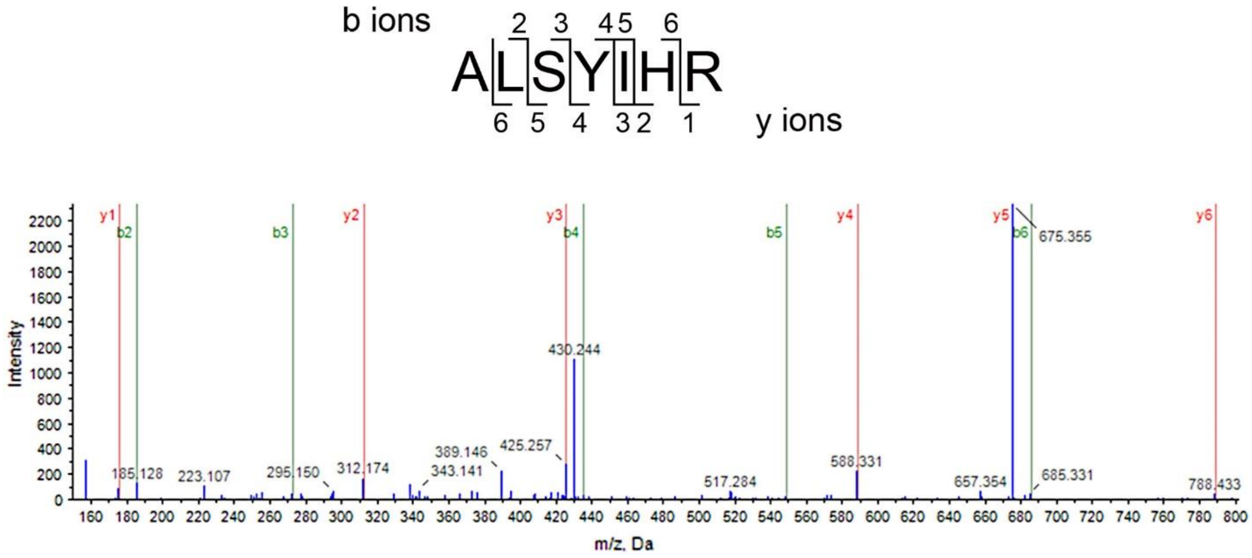

Supplementary Figure 2. MS/MS spectrum of an SK11 peptide identified by pull-down assay of protein extracts from *Arabidopsis* Col-0 wild-type siliques using the recombinant MBP-TTG1 protein. The identified SK11 peptide is shown above the corresponding mass spectrum.

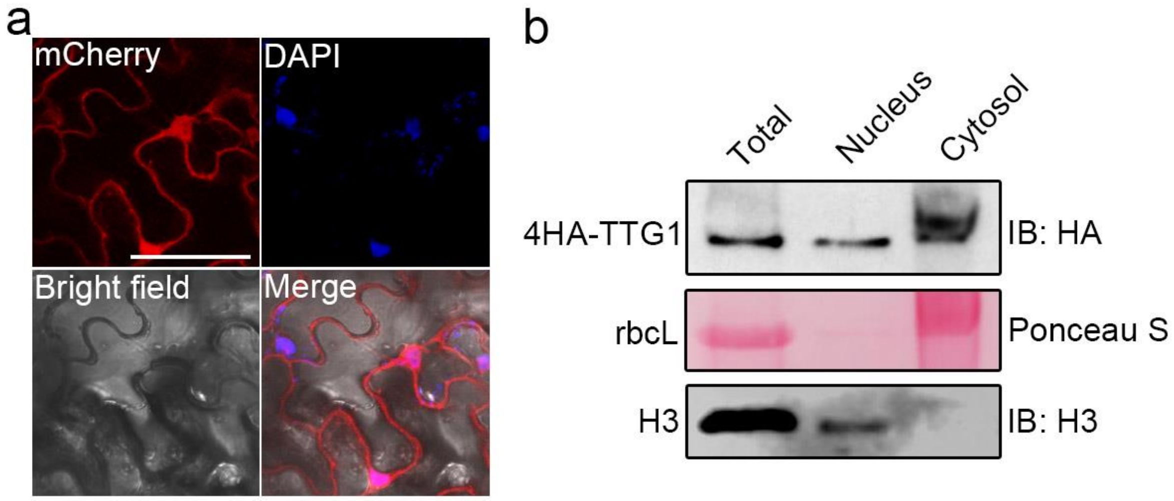

Supplementary Figure 3. TTG1 is localized in both the nucleus and cytosol. (**a**) Subcellular localization of TTG1-mCherry in *N. benthamiana* leaf epidermal cells. DAPI, fluorescence of 4’,6-diamidino-2-phenylindole; Merge, merge of mCherry, DAPI, and bright field. Scale bar, 50 µm. (**b**) Subcellular localization of 4HA-TTG1 in *ttg1-13 g4HA-TTG1* (*#1-1*). Immunoblot analysis using anti-HA antibody (upper panel) reveals the expression of 4HA-TTG1 fusion protein in both cytosolic and nuclear fractions of proteins extracted from siliques 4 days after pollination. The RUBISCO large subunit (rbcL) stained with Ponceau S and immunoblot analysis using anti-histone 3 (H3) were used as the fraction indicators of cytosol and nucleus, respectively. Uncropped original scans of immunoblots are shown in Supplementary Fig. 18.

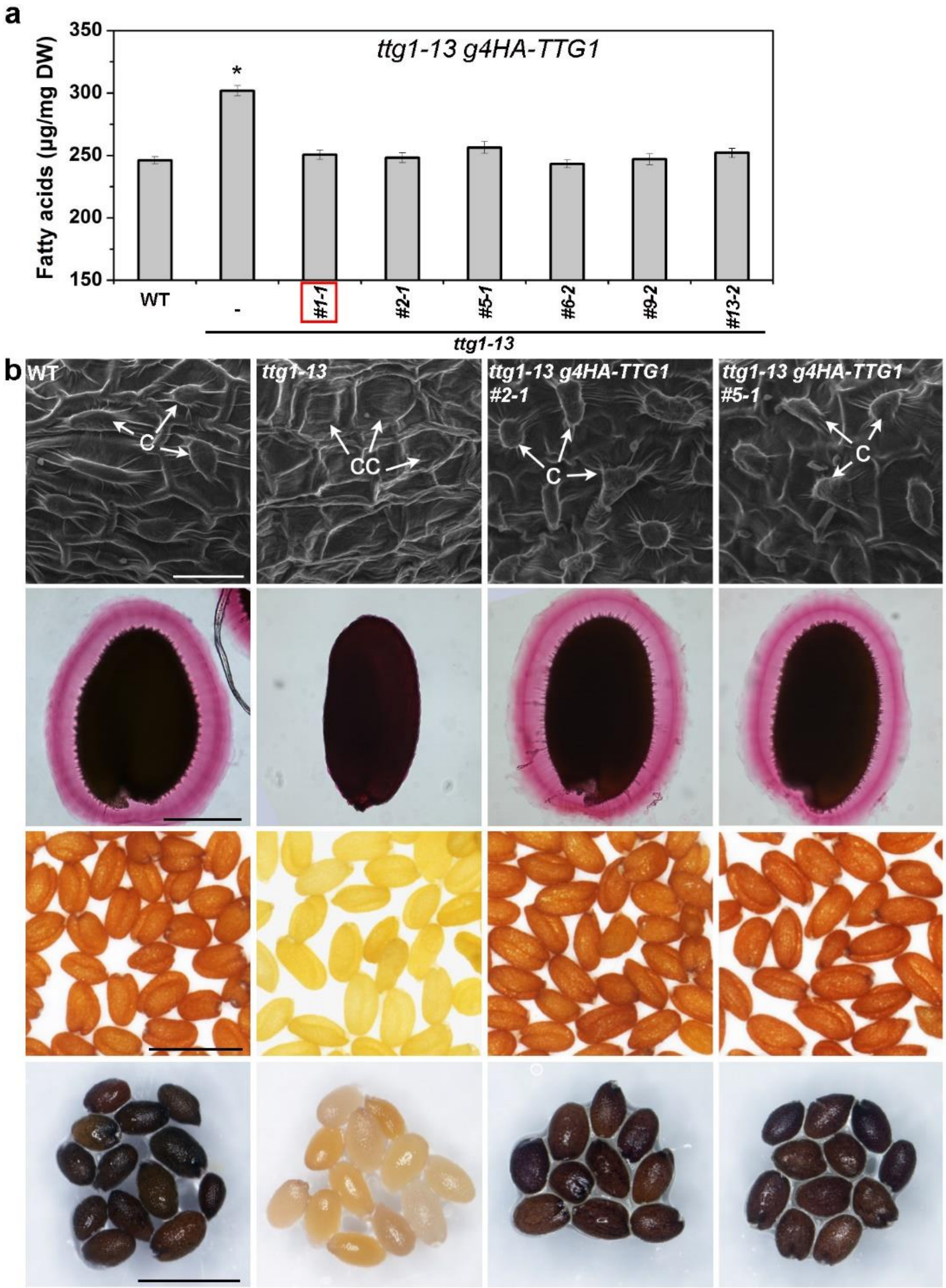

Supplementary Figure 4. Characterization of *ttg1-13 g4HA-TTG1* transgenic plants. (**a**) Measurement of total fatty acid contents of mature seeds of selected *ttg1-13 g4HA-TTG1* transgenic lines that could contain only one T-DNA insertion site based on the segregation ratio. Values are mean ± s.d. of three biological replicates. Asterisk indicates significant difference between wild-type and *ttg1-13* (two-tailed paired Student's *t* test, $^*P < 0.001$). The line highlighted by the red frame was selected for further analysis. (**b**) Examination of seed coat phenotypes of mature *Arabidopsis* seeds in various genetic backgrounds. SEM of seed coat (1st row), seed coat mucilage staining with ruthenium red (2nd row), seed color (3rd row), and seed staining with DMACA (4th row) are shown in panels from top to bottom. Scale bars, 25 µm, 200 µm, 1 mm, and 1 mm (from top to bottom). In SEM panels, "C" indicates columella, while "CC" indicates collapsed columella.

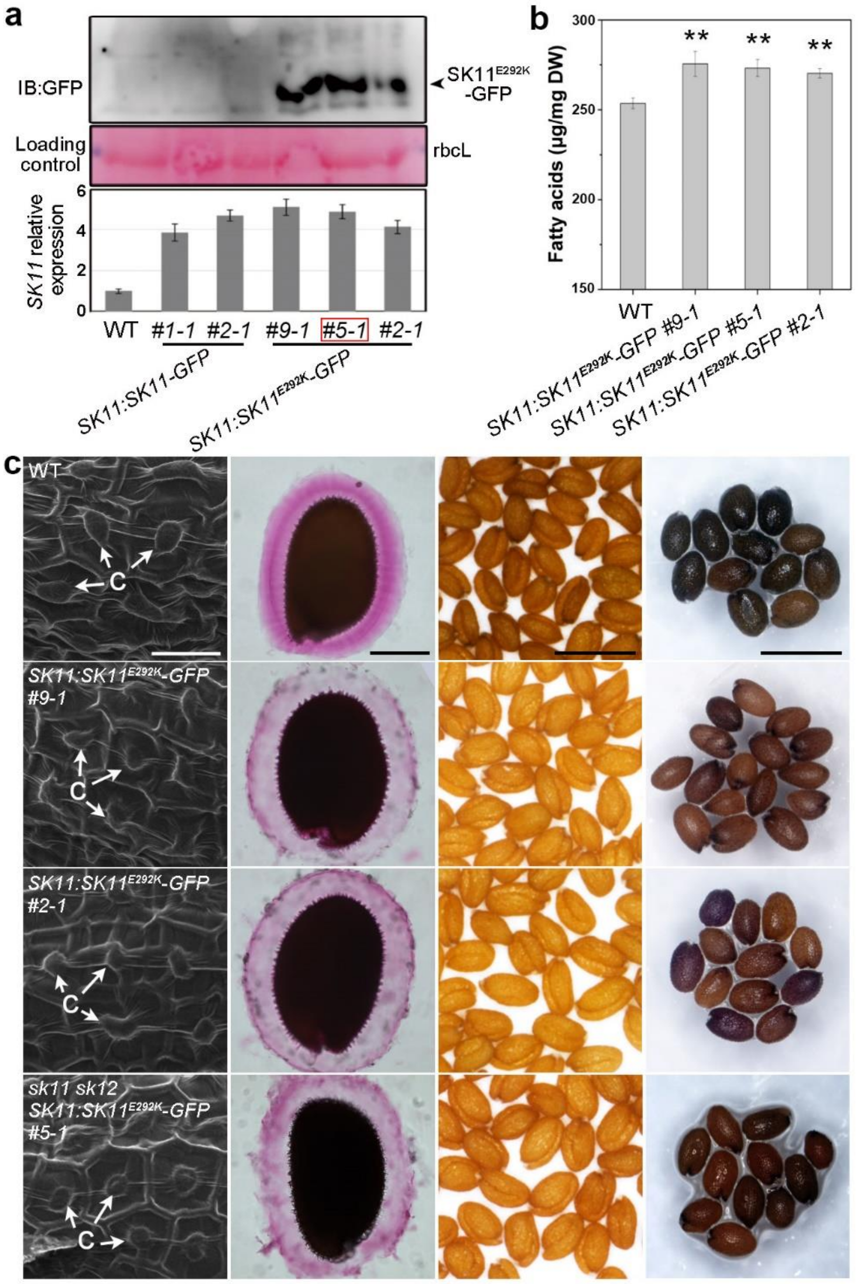

Supplementary Figure 5. Characterization of transgenic plants pertaining to *SK11*. (**a**) Analysis of SK11 protein and gene expression in selected *SK11:SK11-GFP* and *SK11:SK11^{E292K}-GFP* transgenic lines that could contain only one T-DNA insertion site based on the segregation ratio. Total protein and RNA were extracted from siliques 4 days after pollination, and subjected to Western blot analysis using anti-GFP antibody (upper panel) and quantitative real-time PCR analysis (lower panel). Ponceau S staining of RUBISCO large subunit (RbcL) (middle panel) is used as a loading control for Western blot analysis. *SK11* expression values normalized against the expression levels of *U-BOX* are shown relative to the level in wild-type siliques set as 1 (lower panel). Values are mean ± s.d. of three biological replicates. The line highlighted by the red frame was selected for further analysis. Uncropped original scans of immunoblots are shown in Supplementary Fig. 18. (**b**) Measurement of total fatty acid contents of mature seeds in *SK11:SK11^{E292K}-GFP* transgenic lines. Values are mean ± s.d. of three biological replicates. Asterisks indicate significant differences between wild-type and transgenic lines (two-tailed paired Student's *t* test, $^{**}P < 0.001$). (**c**) Examination of seed coat phenotypes of mature seeds in various genetic backgrounds. Scanning electron microscopy (SEM) of seed coat (1st column), seed coat mucilage staining with ruthenium red (2nd column), seed color (3rd column), and seed staining with DMACA (4th column) are shown in panels from left to right. Scale bars, 25 µm, 200 µm, 1 mm, and 1 mm (from left to right). In SEM panels, "C" indicates columella.

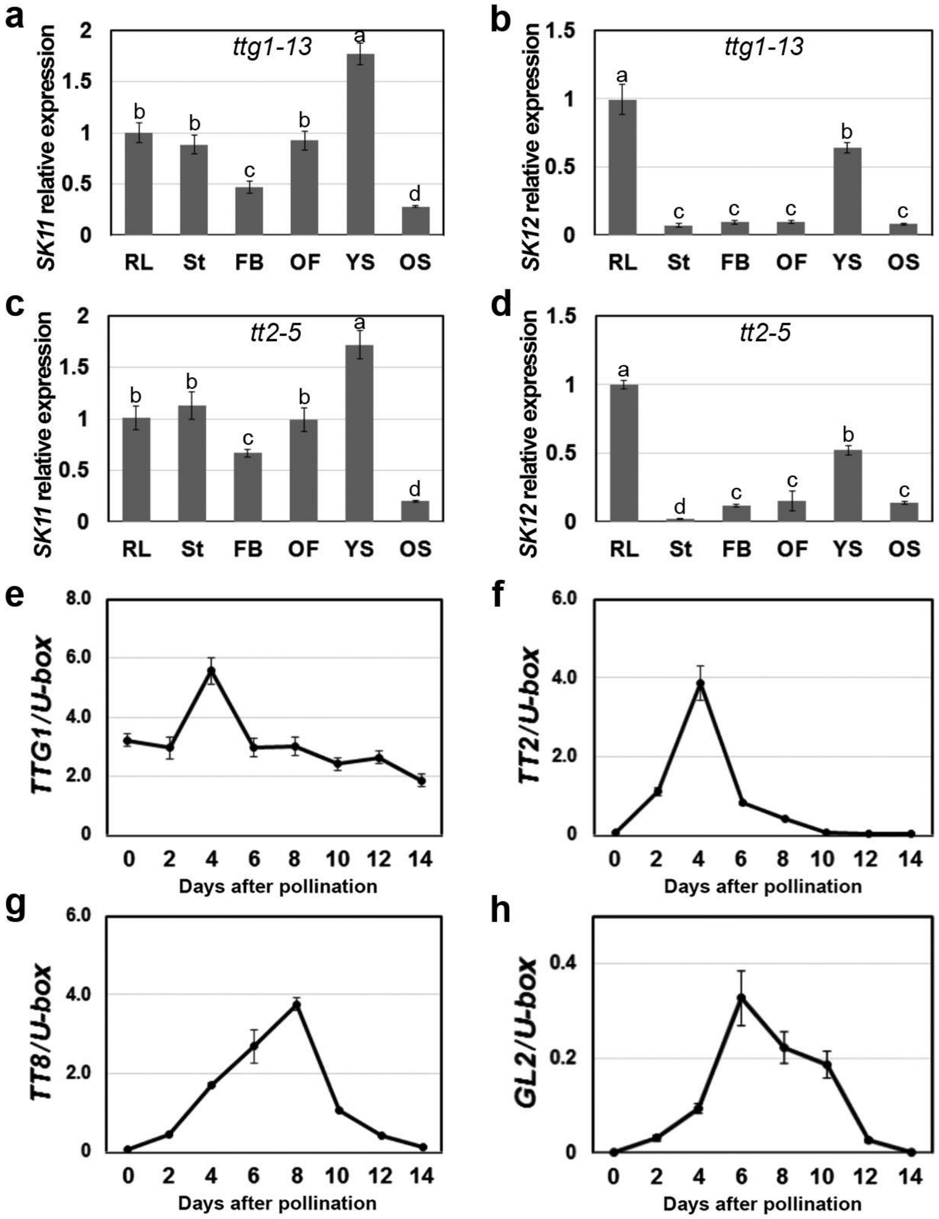

Supplementary Figure 6. Quantitative real-time PCR analysis of *SK11* and *SK12* expression in *ttg1-13* and *tt2-5*, and expression of *TTG1*, *TT2*, *TT8* and *GL2* in developing seeds of wild-type plants. (**a**,**b**) Expression of *SK11* (**a**) and *SK12* (**b**) in aerial tissues of adult *ttg1-13* plants. (**c**,**d**) Expression of *SK11* (**c**) and *SK12* (**d**) in aerial tissues of adult *tt2-5* plants. RL, rosette leaf; St, stem; FB, flower bud; OF, open flower; YS, young silique (before 7 days after pollination); OS, old silique (7-12 days after pollination). Expression values normalized against the expression levels of *U-BOX* are shown relative to the level in rosette leaves set as 1. Values are mean ± s.d. of three biological replicates. Samples in each panel indicated with the same letter (a–d) above the bars are not significantly different. *p* values were determined by global one-way ANOVA with Holm-Bonferroni post hoc tests ($\alpha = 0.05$). (**e-h**) Expression of *TTG1* (**e**), *TT2* (**f**), *TT8* (**g**) and *GL2* (**h**) in developing seeds of wild-type plants. Results were normalized against the expression levels of *U-BOX* as an internal control. Values are mean ± s.d. of three biological replicates.

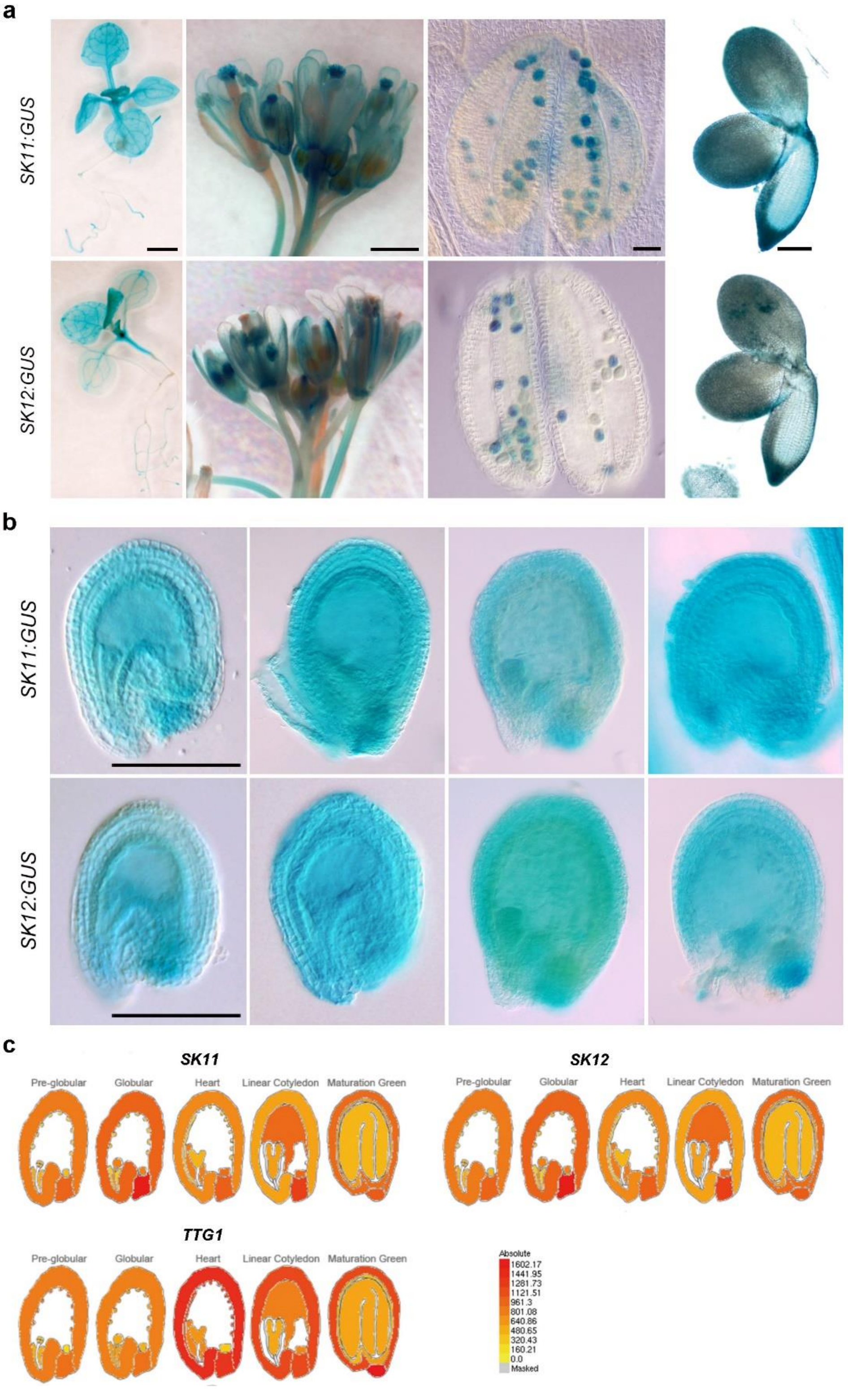

Supplementary Figure 7. Expression patterns of *SK11*, *SK12* and *TTG1*. (**a**) Representative GUS staining of *SK11:GUS* and *SK12:GUS* in vegetative seedlings (1st column), flowers (2nd column), anthers (3rd column), and mature embryos (last column). Scale bars from left to right, 1 mm, 1 mm, 0.1 mm, 0.1 mm. (**b**) GUS staining of several *SK11:GUS* (upper panels) and *SK12:GUS* (lower panels) seeds 2 days after pollination. Scale bars, 200 µm. (**c**) Expression patterns of *SK11*, *SK12* and *TTG1* in various stages of seed development from the *Arabidopsis* eFP browser.

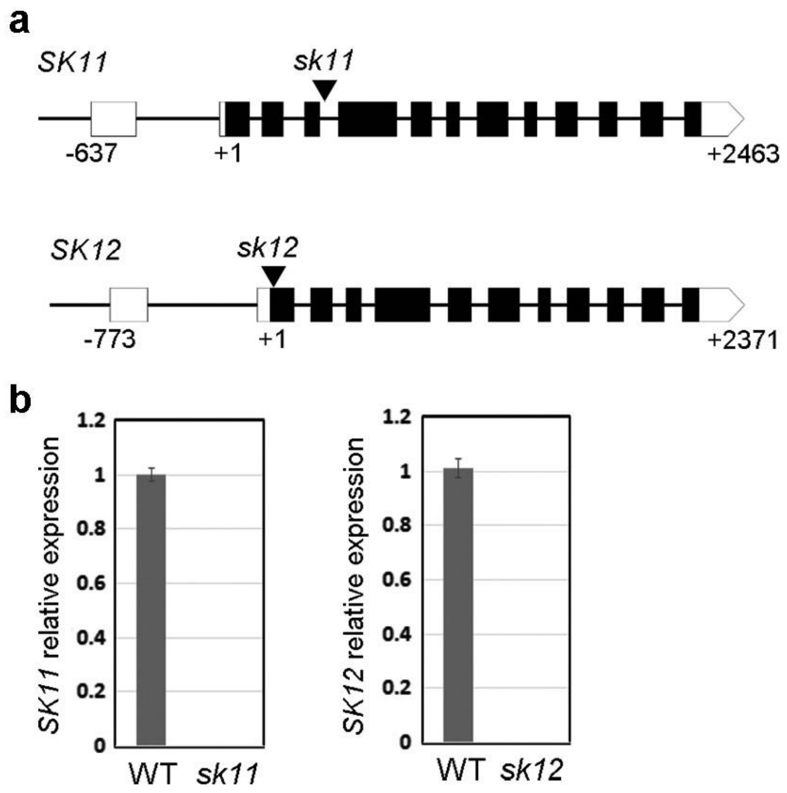

Supplementary Figure 8. Characterization of T-DNA insertion mutants of *SK11* and *SK12.* (**a**) Schematic diagrams show the T-DNA insertion sites in *sk11* (SALK_014382) and *sk12* (CS332559) mutants. The coding and untranslated regions are indicated by black and white boxes, respectively, while other genomic regions are indicated by black lines. The first nucleotide of the translation start codon is assigned the +1 position, and all other sequences are numbered relative to this site. Arrowheads indicate the T-DNA insertion sites. (**b**) Quantitative real-time PCR analysis of *SK11* (left panel) and *SK12* (right panel) expression in *sk11* and *sk12* siliques 4 days after pollination, respectively. Gene expression values normalized against the expression levels of *U-BOX* are shown relative to the level in wild-type siliques set as 1. Values are mean ± s.d. of three biological replicates.

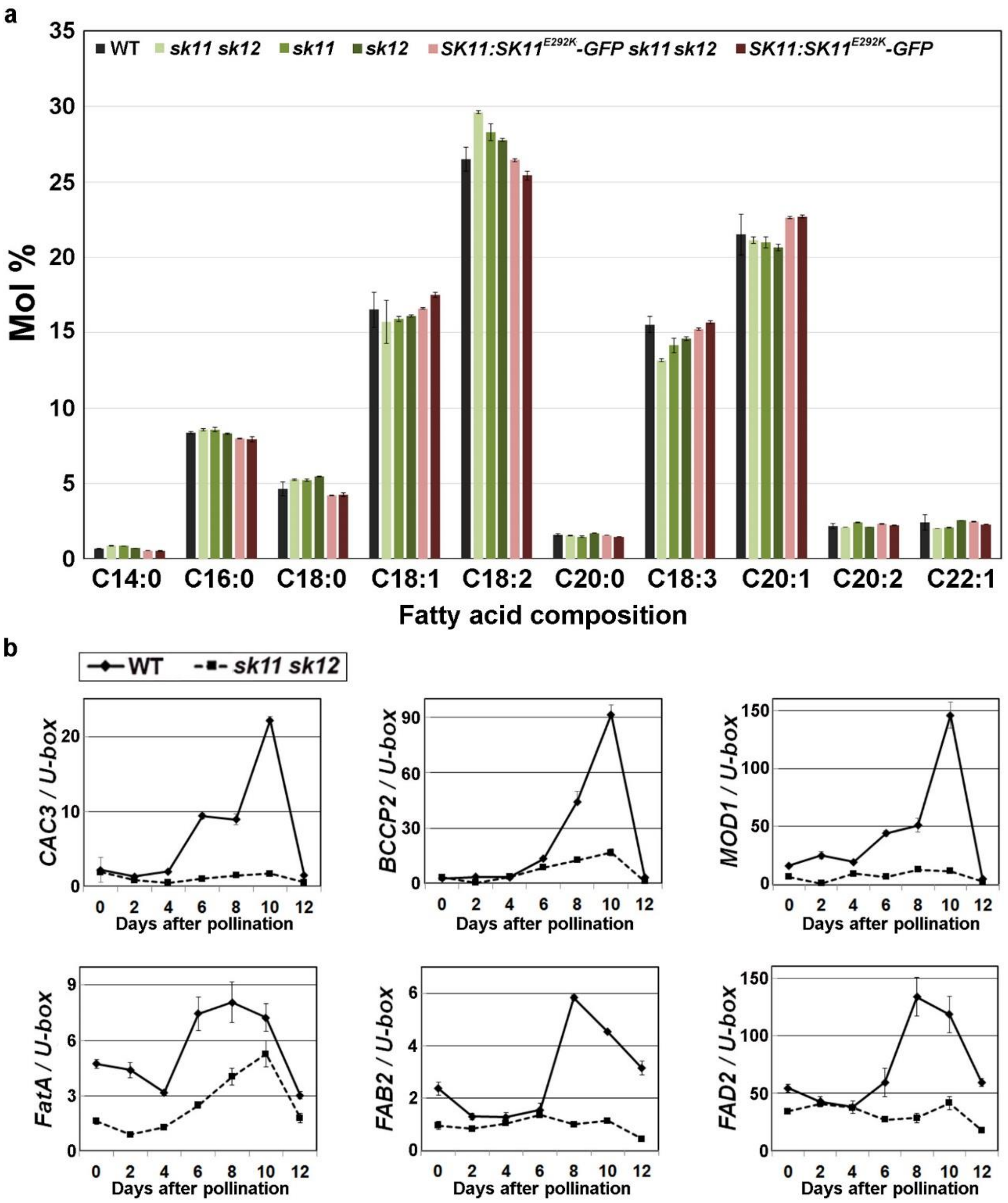

Supplementary Figure 9. Characterization of *sk11 sk12* seeds. (**a**) Comparison of fatty acid composition (mol%) of total lipid extracts from mature seeds of various genetic background by GC-MS. Values are mean ± s.d. of three biological replicates. (**b**) Quantitative real-time PCR analysis of expression of fatty acid biosynthetic genes in developing seeds of wild-type and *sk11 sk12* plants. Results were normalized against the expression levels of *U-BOX* as an internal control. Values are mean ± s.d. of three biological replicates.

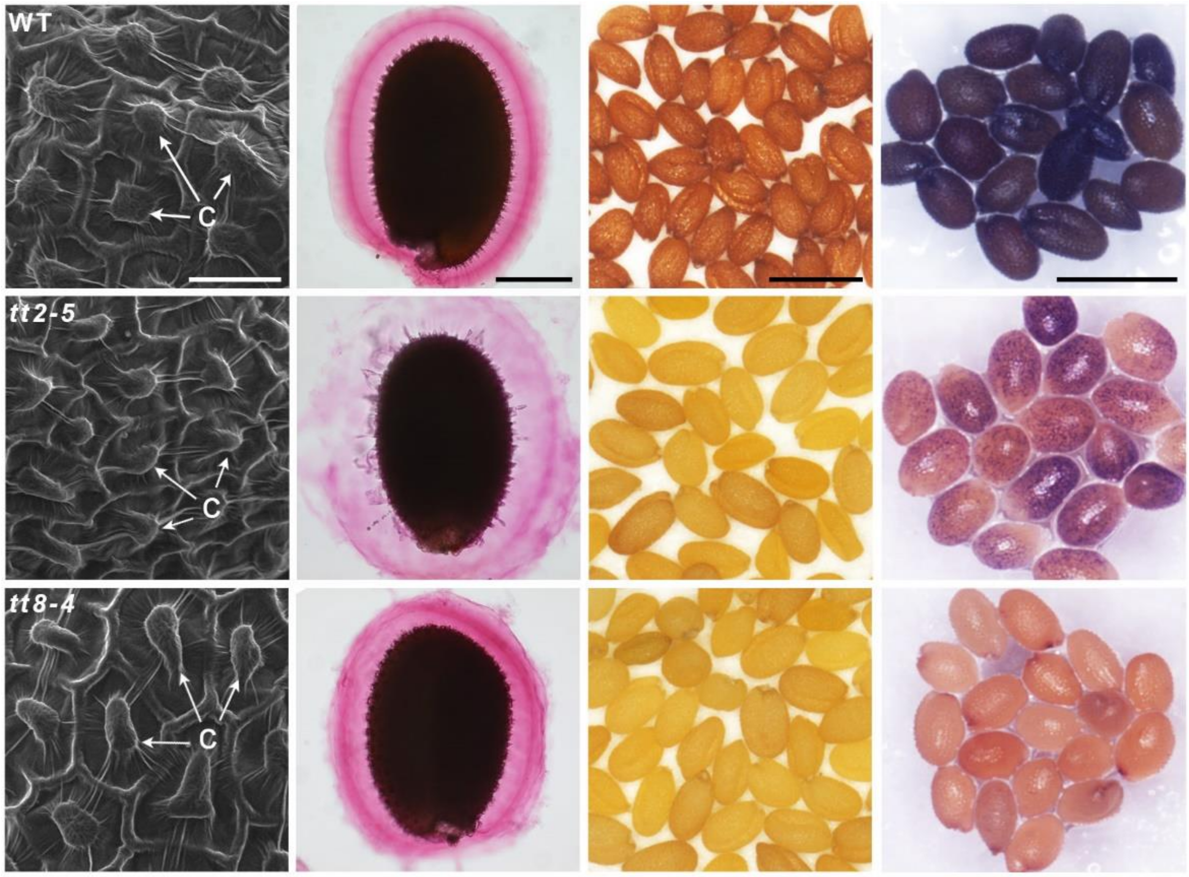

Supplementary Figure 10. Examination of seed coat phenotypes of mature seeds of *tt2-5* and *tt8-4*. SEM of seed coat (1st column), seed coat mucilage staining with ruthenium red (2nd column), seed color (3rd column), and seed staining with DMACA (4th column) are shown in panels from left to right. Scale bars, 25 µm, 200 µm, 1 mm, and 1 mm (from left to right). In SEM panels, “C” indicates columella.

a

P-site by SK11	pS85	pS215	pS252
Hits	3	2	1

b

P-site by SK12	pS85	pS88	pT169	pS215
Hits	5	1	1	4

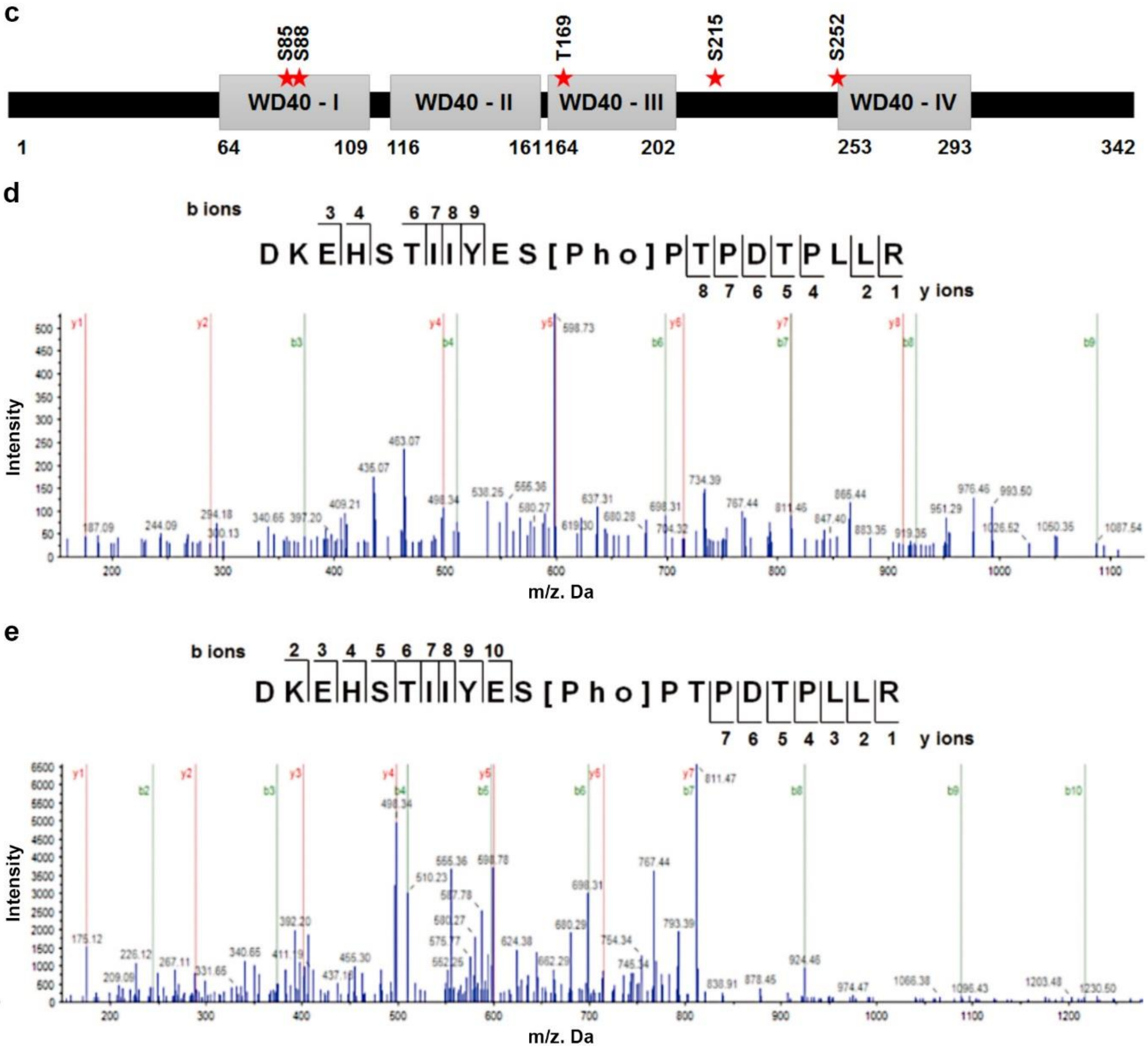

Supplementary Figure 11. Identification of SK11- and SK12-mediated phosphorylation sites on TTG1. (**a**,**b**) Peptide hits for potential TTG1 phosphorylation sites identified by LC-MS/MS analysis of phosphorylated products mediated by GST-SK11 (**a**) or GST-SK12 (**b**) in *in vitro* kinase assay shown in Fig. 4a. (**c**) Schematic diagram of the sites on TTG1 protein potentially phosphorylated by SK11 and SK12. Numbers indicate positions of amino acid residues. (**d,e**) MS/MS spectrum of the TTG1 peptide containing serine 215 phosphorylated by SK11 (**d**) or SK12 (**e**). The TTG1 phosphor-peptide containing residues surrounding serine 215 is shown above the corresponding mass spectrum.

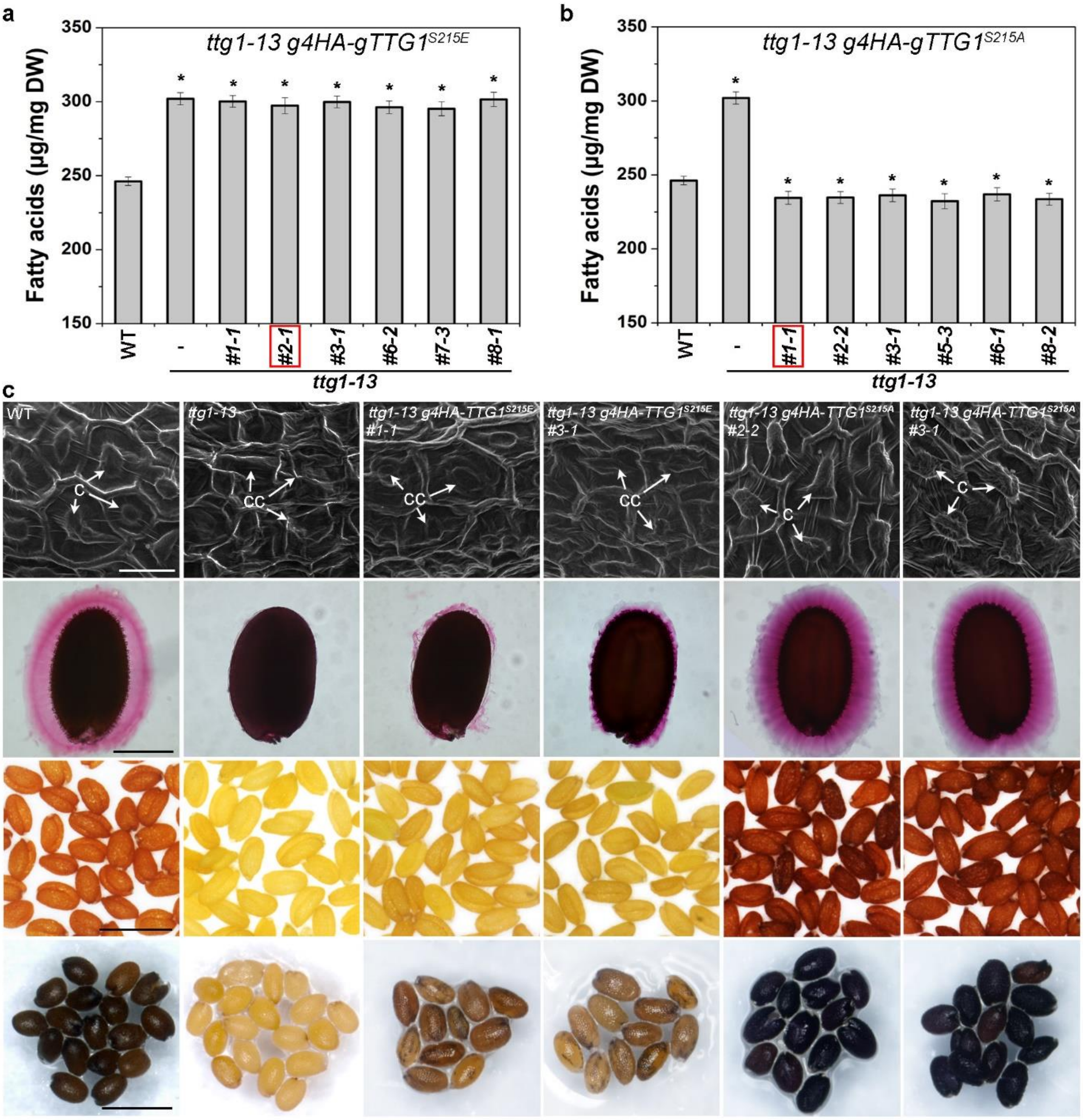

Supplementary Figure 12. Characterization of transgenic plants harboring TTG1 variants. (**a**,**b**) Measurement of total fatty acid contents of mature seeds of selected *g4HA-TTG1*^{S215E} (**a**) and *ttg1-13 g4HA-TTG1*^{S215A} (**b**) transgenic lines that could contain only one T-DNA insertion site based on the segregation ratio.. Values are mean ± s.d. of three biological replicates. Asterisks indicate significant differences between wild-type and other samples (two-tailed paired Student's *t* test, *$P < 0.001$). (**e**) Examination of seed coat phenotypes of mature *Arabidopsis* seeds in various genetic backgrounds. SEM of seed coat (1st row), seed coat mucilage staining with ruthenium red (2nd row), seed color (3rd row), and seed staining with DMACA (4th row) are shown in panels from top to bottom. Scale bars, 25 µm, 200 µm, 1 mm, and 1 mm (from top to bottom). In SEM panels, "C" indicates columella, while "CC" indicates collapsed columella.

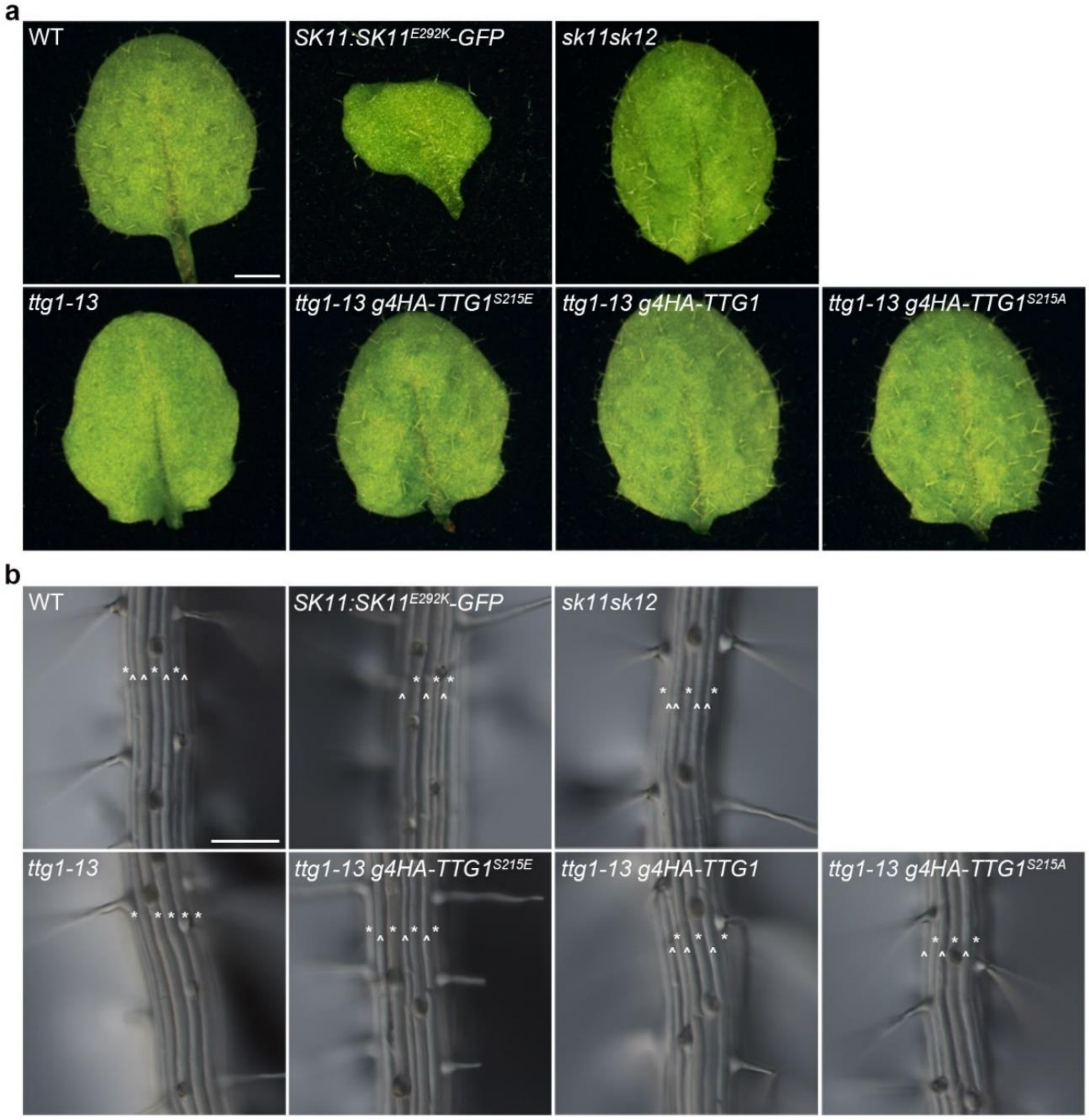

Supplementary Figure 13. SK11/12 and phosphorylation of TTG1 at serine 215 do not affect the formation of trichomes and root hairs. (**a**) Examination of leaf trichome phenotype in *Arabidopsis* plants in various genetic backgrounds. *ttg1-13* develops leaves without trichomes, whereas leaves in other genetic backgrounds all produce trichomes. Scale bar, 1 mm. (**b**) Examination of root hair formation in *Arabidopsis* plants in various genetic backgrounds. *ttg1-13* generates root hair cells adjacent to one another, whereas root hair cells in other genetic backgrounds are separated by either one or two non-hair cells. "*" indicate a hair cell, while " ^ " indicate a non-hair cell. Scale bar, 100 µm.

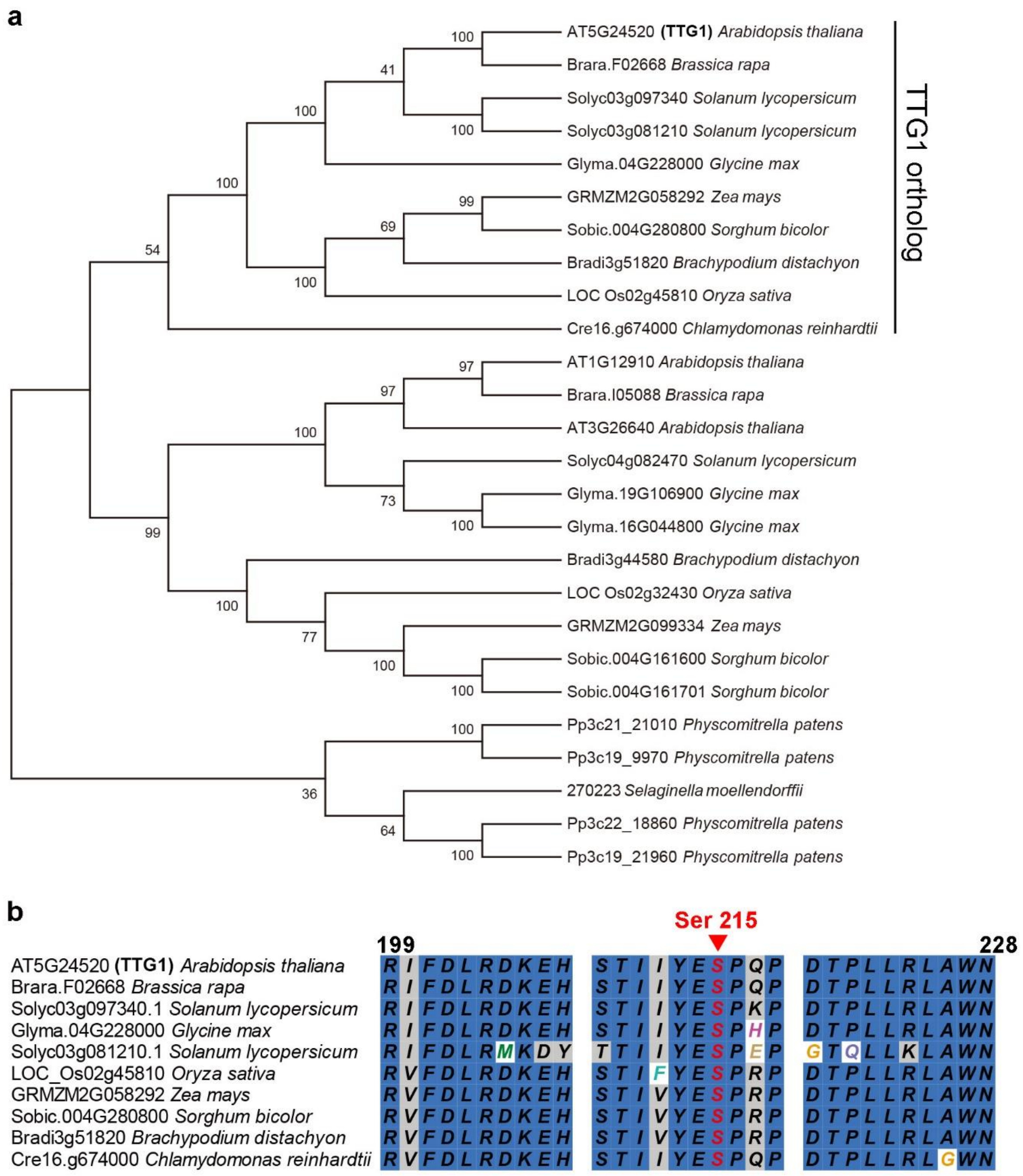

Supplementary Figure 14. Ser215 of TTG1 is conserved among TTG1 orthologs in various plant species. (**a**) Phylogenetic analysis of TTG1 homologs in various plant species. The neighbor-joining (NJ) phylogeny was performed using MEGA 7.0.14 with 1000 replicates. The orthologs of TTG1 are grouped from other homologs. (**b**) Alignment of amino acid residues containing Ser215 among various TTG1 orthologs. The numbers above the alignment indicate the positions of amino acids in *Arabidopsis* TTG1, while the conserved serines corresponding to Ser215 of TTG1 are highlighted in red. This alignment was generated using the MAFFT version 6 with G-INS-i strategy.

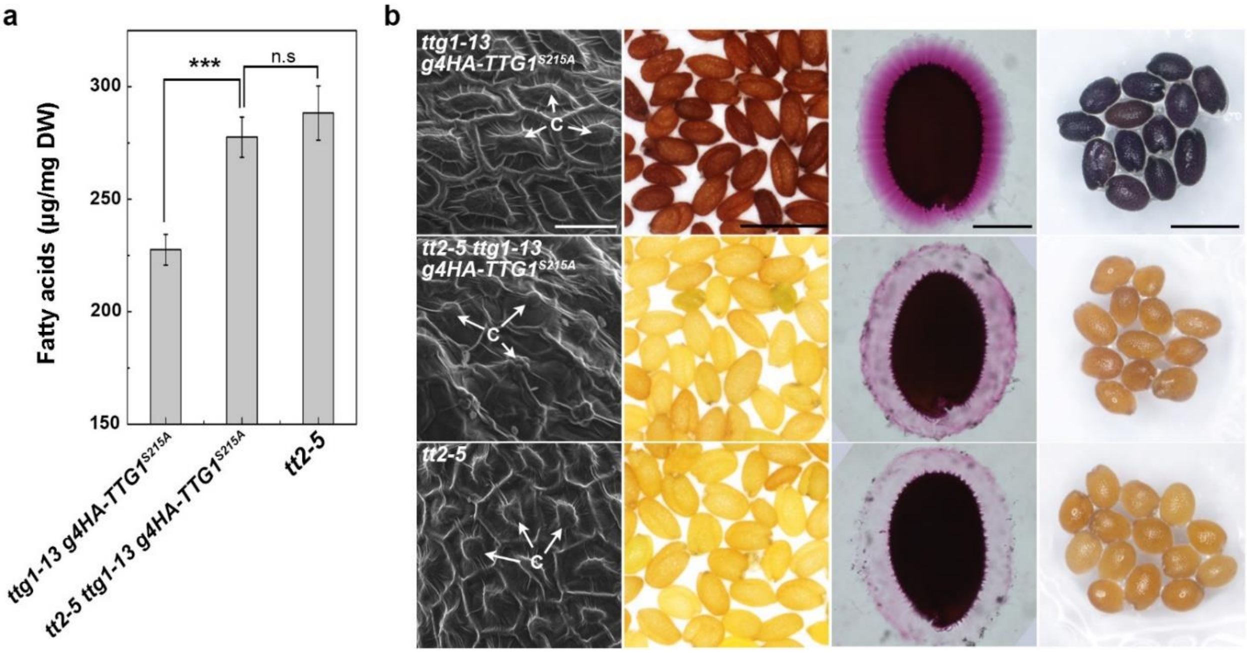

Supplementary Figure 15. Effects of 4HA-TTG1^{S215A} on seed phenotypes are dependent on TT2. (**a**) Measurement of total fatty acid contents of mature seeds in *ttg1-13 g4HA-TTG1^{S215A}*, *tt2-5 ttg1-13 g4HA-TTG1^{S215A}* and *tt2-5*. Values are mean ± s.d. of three biological replicates. Asterisks and n.s indicate significant and non-significant differences between specified samples, respectively (two-tailed paired Student's *t* test, ***$P < 0.001$. (**b**) Examination of seed coat phenotypes of mature seeds of *ttg1-13 g4HA-TTG1^{S215A}*, *tt2-5 ttg1-13 g4HA-TTG1^{S215A}* and *tt2-5*. SEM of seed coat (1st column), seed color (2nd column), seed coat mucilage staining with ruthenium red (3rd column), and seed staining with DMACA (4th column) are shown in panels from left to right. Scale bars, 25 μm, 1 mm, 200 μm, and 1 mm (from left to right). In SEM panels, "C" indicates columella.

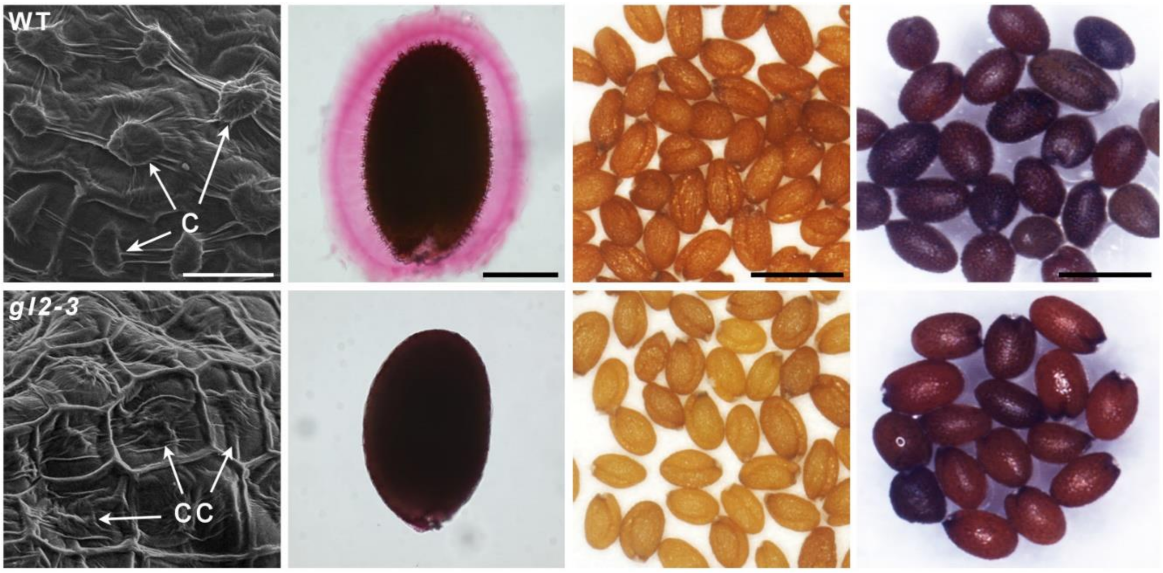

Supplementary Figure 16. Examination of seed coat phenotypes of mature *gl2-3* seeds. SEM of seed coat (1st column), seed coat mucilage staining with ruthenium red (2nd column), seed color (3rd column), and seed staining with DMACA (4th column) are shown in panels from left to right. Scale bars, 25 µm, 200 µm, 1 mm, and 1 mm (from left to right). In SEM panels, "C" indicates columella, while "CC" indicates collapsed columella.

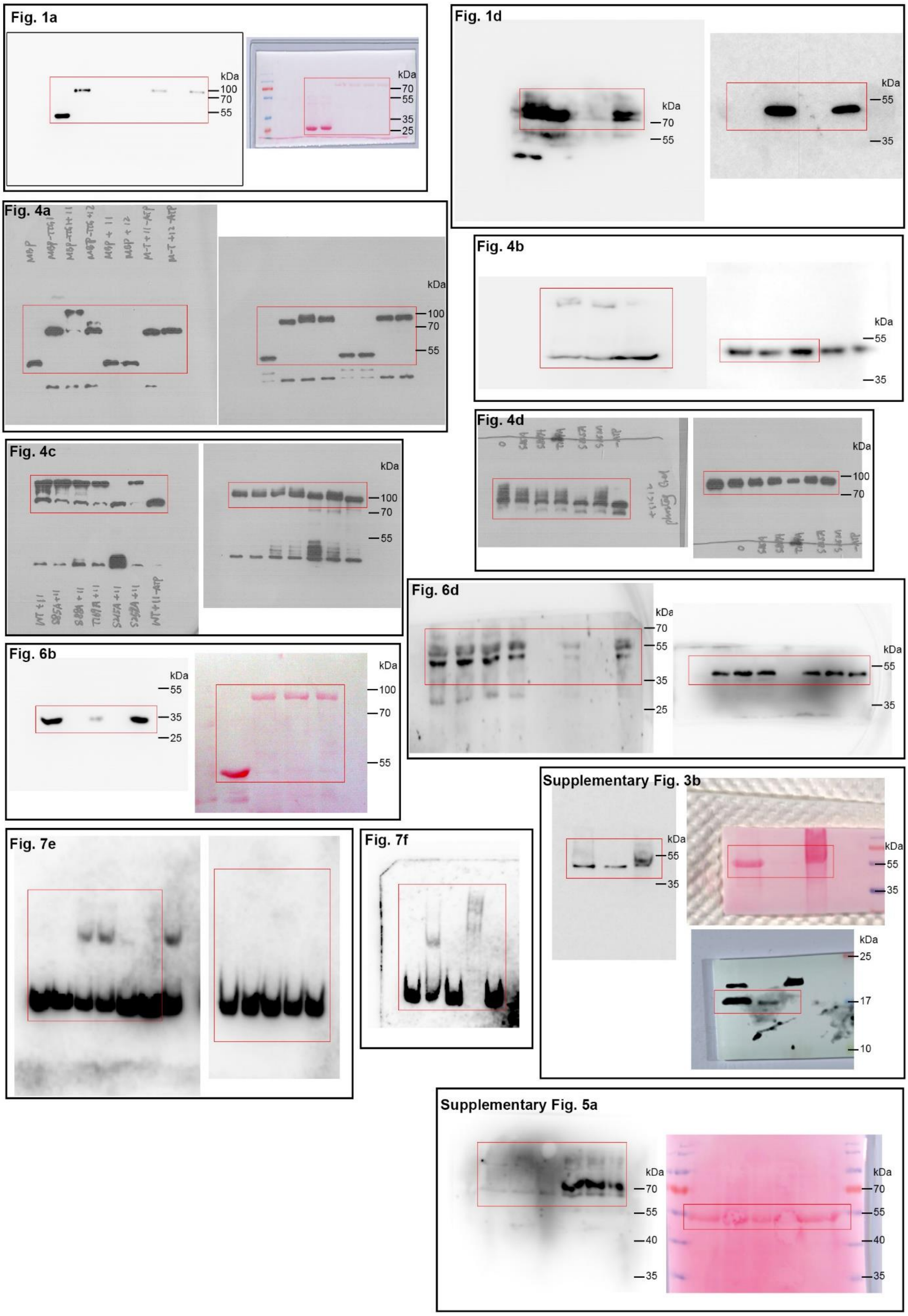

Supplementary Figure 17. Representative uncropped scanned images of immune-blots. Red frames indicate selected portions presented in various figures in this paper.

Supplementary Table 1. Primers used in this study

Primers for quantitative real-time PCR

Gene name	Primers
SK11	5’-CCTAATCTACGAAGTGCCGCTCTC-3’
	5’-ACGTGCGTTTGGGTCTCTTAAC-3’
SK12	5’-AGCTGTTGATTTGGTCTCAAGGC-3’
	5’-ATGGGTGGACCAATGAATCAAGAG-3’
SK13	5’-TGCTGAATTGCTTCTGGGACAGC-3’
	5’-TCAACTAGCTGGTCAACTCCACTC-3’
BIN2	5’-GTCTAAGATGCACAGCGCTCGAAG-3’
	5’-AAAGGCCGTCCATTTGGTAAACG-3’
TTG1	5’-AGCTCCTTAGAGTTTGAGGTGCAG-3’
	5’-GCGCAAACCAAACCTACTTACGC-3’
TT2	5’-ACTCTCCCTAACCAAGCTGGTCTC-3’
	5’-CCCGGTCTTAGGTAGTTCTTCCAC-3’
TT8	5’-TGTCCTCAACAACGGGTCTTGG-3’
	5’-GCTGGTTGAGTTGTCTTCCTCGTC-3’
GL2	5’-GCAACTCAGTGGCAATCCAGAC-3’
	5’-TGTCTTGCAGCACCCATATGCTC-3’
U-BOX	5’-TCTTCTTCTGCTACATCTACTCTC-3’
	5’-AGTGTGTGAACCCGTGAAC-3’

Primers for constructs used for plant transformation

Construct name	Primers
g4HA-TTG1 & *g4HA-TTG1^{S215A}* & *g4HA-TTG1^{S215E}*	5’-GTCGAGCGCGTCGACTGTGTTAAACTTTCTTTTTTGTCTTCTTATGTG-3’
	5’-CATCGTATGGGTAACTAGACATGGAAATGTGTGGTGCTGATTCGAT-3’
	5’-TCAGCACCACACATTTCCATGTCTAGTTACCCATACGATGTTCC-3’
	5’-GGAGCTGAATTATCCATTCTAGTAGCGTAATCTGGAACGT-3’
	5’-TTCCAGATTACGCTACTAGAATGGATAATTCAGCTCCAGATTCG-3’
	5’-AGACTAGTGGATCCCCCGGGAAATTCAATTTAAATCTGTGTCAAATAAAC-3’

	5’-TCTACAATCATTTACGAGGCTCCTC-3’<br>5’-GCGTATCAGGCTGAGGAGCCTC-3’<br>5’-TCTACAATCATTTACGAGGAGCCTC-3’<br>5’-GCGTATCAGGCTGAGGCTCCTC-3’
SK11:GUS	5’-GTCGACGGTATCGATAAGCTTTTCTCATTAGTCTGTTATCGTCGG-3’<br>5’-CGGGGATCCTCTAGAGTCGACTTTTCAGGCTACAAAACTCTTCAAAC-3’
SK12:GUS	5’-GCTATGACCATGATTACGCCAAGCTTGGAGAAGCAAAACCTTAACAATG-3’<br>5’-CGGGGATCCTCTAGAGTCGACCCCAAGATTAAAACAGAAGAATTTG-3’
SK11:SK11-GFP<br>&<br>*SK11:SK11*^{E292K}*-GFP*	5’-NCGAATTCTTCTCATTAGTCTGTTATCGTCGG-3’<br>5’-GGGGTACCTTTTCAGGCTACAAAACTCTTCAAAC-3’<br>5’-NCGGGATCCATGGCGTCAGTGGGTATAGCTC-3’<br>5’-NCCTGTCGACCAAACCGAGCCAAGGACACTG-3’<br>5’-GGTCTTGGGAACGCCTACTAGAAAA-3’<br>5’-TGCACTTGATTTCTTTTCTAGTAGGCG-3’
35S:TT2-GFP	5’-NGCTCTAGAATGGGAAAGAGAGCAACTACTAGTG-3’<br>5’-NGCTCTAGATTAACAAGTGAAGTCTCGGAGCCAA-3’

Primers for constructs used for yeast two-hybrid assays

**Construct name**	**Primers**
AD-TTG1<br>&<br>*AD-TTG1*^{S215A}<br>&<br>*AD-TTG1*^{S215E}	5’-NGCGAATTCATGGATAATTCAGCTCCAGAT-3’<br>5’-CCATCGATCTAAACTCTAAGGAGCTGCATTTTG-3’<br>5’-TCTACAATCATTTACGAGGCTCCTC-3’<br>5’-GCGTATCAGGCTGAGGAGCCTC-3’<br>5’-TCTACAATCATTTACGAGGAGCCTC-3’<br>5’-GCGTATCAGGCTGAGGCTCCTC-3’
BD-SK11	5’-NTCCATATGATGGCGTCAGTGGGTATAGCTC-3’<br>5’-NCCGTCGACCAAACCGAGCCAAGGACACTG-3’
BD-SK12	5’-NGCGAATTCATGGCCTCGGTGGGCATAGAG-3’<br>5’-NCCGTCGACCAAACTGAGCCACGGACATTGCT-3’
BD-TT2	5’-NCCTGTCGACATATGGGAAAGAGAGCAACTACTAGTG-3’<br>5’-NAACTGCAGACAAGTGAAGTCTCGGAGCCAA-3’
BD-TT8	5’-NCCTGTCGACATATGGATGAATCAAGTATTATTCCG-3’

	5’-NAACTGCAGTAGATTAGTATCATGTATTATGACTTGG-3’

Primers for constructs used for expressing recombinant proteins

Construct name	Primers
TT2-His	5’-ACCAAGCTTATGGGAAAGAGAGCAACTACTAGTG-3’
	5’-CCGCTCGAGACAAGTGAAGTCTCGGAGCCAA-3’
MBP-TT2	5’-NCCTGTCGACATGGGAAAGAGAGCAACTACTAGTG-3’
	5’-NAACTGCAGACAAGTGAAGTCTCGGAGCCAA-3’
MBP-TTG1	5’-NCCTGTCGACATGGATAATTCAGCTCCAGATTCG-3’
&	5’-NAACTGCAGAACTCTAAGGAGCTGCATTTTGTTAG-3’
MBP-TTG1^{S85A}	5’-TCTACAATCATTTACGAGGAGCCTC-3’
&	5’-GCGTATCAGGCTGAGGCTCCTC-3’
MBP-TTG1^{S88A}	5’-TTCGCTCCTCCTTCTCTCCGTCGTC-3’
&	5’-AGGACGACGGAGAGAAGGAGGAGCGAAC-3’
MBP-TTG1^{T169A}	5’-GTCCTCCTGCTCTCCGTCGTCCTT-3’
&	5’-CGGAGGAAGGACGACGGAGAGCAG-3’
MBP-TTG1^{S215A}	5’-AGAAGTCTGTTGTTGAGGCTCAGCTTATAG-3’
&	5’-TTATCATGAGCTATAAGCTGAGCCTC-3’
MBP-TTG1^{S252A}	5’-TCTACAATCATTTACGAGGCTCCTC-3’
	5’-GCGTATCAGGCTGAGGAGCCTC-3’
	5’-GGTTGTGATTCTCGATATTCGTGCG-3’
	5’-GGCGCACGAATATCGAGAATCAC-3’
GST-SK11	5’-NCGGGATCCATGGCGTCAGTGGGTATAGCTC-3’
	5’-NCCTGTCGACCAAACCGAGCCAAGGACACTG-3’
GST-SK12	5’-NCGGGATCCATGGCCTCGGTGGGCATAGAG-3’
	5’-NCCTGTCGACCAAACTGAGCCACGGACATTGCT-3’

Primers for constructs used for BiFC assays

Construct name	Primers
nYFP-SK11	5’-NCGGGATCCATGGCGTCAGTGGGTATAGCTC-3’
	5’-NCCTGTCGACTCACAAACCGAGCCAAGGACACTG-3’
nYFP-SK12	5’-NCGGGATCCATGGCCTCGGTGGGCATAGAG-3’
	5’-NCCTGTCGACTCACAAACTGAGCCACGGACATTGCT-3’
TTG1-cYFP	5’-CATGGTACCATGGATAATTCAGCTCCAGATTCG-3’
&	5’-GCATCTAGAAACTCTAAGGAGCTGCATTTTGTTAG-3’
TTG1^{S215A}*-cYFP*	5’-TCTACAATCATTTACGAGGCTCCTC-3’
&	5’-GCGTATCAGGCTGAGGAGCCTC-3’

TTG1^{S215E}-cYFP	5'-TCTACAATCATTTACGAGGAGCCTC-3'<br>5'-GCGTATCAGGCTGAGGCTCCTC-3'
nYFP-TT2	5'-NGCTCTAGAATGGGAAAGAGAGCAACTACTAGTG-3'<br>5'-NGCTCTAGATTAACAAGTGAAGTCTCGGAGCCAA-3'
nYFP-TT8	5'-NCGGGATCCATGGATGAATCAAGTATTATTCCG-3'<br>5'-NCCTGTCGACTCATAGATTAGTATCATGTATTATGACTTGG-3'

Primers for ChIP assays

Number	Primers
ChIP-GL2-P1	5'-AGCGGCTTTGGTCTGAATTTTTT-3'<br>5'-GTTAGCGTCTTAGCGAGCGTGC-3'
ChIP-GL2-P2	5'-GTAAGCAAAATGTGTAAGATTCAAGGT-3'<br>5'-ACAAAAGATGGATAGTTAGTTGGAGAG-3'
ChIP-GL2-P3	5'-ATCATCACCATATTCCATTTTTGG-3'<br>5'-GATCCCTTTTAATTCACGTCCTTAC-3'
ChIP-GL2-P4	5'-AGTAGATAGATAGAGCAAAAGGAGAGG-3'<br>5'-ATATAATGGTATAACTGTAGCAGATGATT-3'
ChIP-GL2 P5	5'-TCCTAATACTGCTACGTACATACCCC-3'<br>5'-CTAAATTGCTTGAGACTTGTCCTCTT-3'
ChIP-GL2-P6	5'-TAAGGAGGGAAGAAGAAAGCAGAAA-3'<br>5'-AAGGGTATAAAAACAAAAAAGACGAACT-3'
ChIP-GL2-P7	5'-GTAATCTATAGCAACGCCATTATGTAC-3'<br>5'-ATAAAGGATTGAGAAGAACTTGAACA-3'
ChIP-GL2-P8	5'-ACCTAAAAGTCAAGAGCAGTAGAGAA-3'<br>5'-TCTTCTCCTCGCACTCCTTCTT-3'
ChIP-GL2-P9	5'-AGAAGGAGTGCGAGGAGAAGAGGG-3'<br>5'-GAGAGGGCTGGAGAGGAGAAAAAGT-3'
ChIP-GL2-P10	5'-AACAAGGGCACTAATAAGAGAAAGA-3'<br>5'-TTAAGTGTTTTAATTTGTGAAGCATAAT-3'
ChIP-GL2-P11	5'-CTTGGACGCACTCCCTATCCCCTG-3'<br>5'-GCAAAGACGCCCGTGTAGAAATCG-3'
ChIP-GL2-P12	5'-AGATGGCTCAGAGAATGACACAAA-3'<br>5'-AACAGGTAACCACAGCGAAGAAG-3'
ChIP-GL2-P13	5'-CCAATCCTATTATTATCCAACACCTTT-3'<br>5'-TTTGCTCTTACTTCATCTTCACCTCC-3'

Primers for producing EMSA probes

Probe name	Primers
Competitor	5’-ATAATATATAAGGAAGAAGGAGTGCGA-3’
	5’-AATATATGTTTATGTGAGAGCTAGCAAGT-3’
Native probe	5’-Bio-ATAATATATAAGGAAGAAGGAGTGCGA-3’
	5’-Bio-AATATATGTTTATGTGAGAGCTAGCAAGT-3’
Mutated probe	5’-Bio-ATAATATATAAGGAAGAAGGAGTGCGA-3’
	5’-Bio-AATATATGTTTATGTGAGAGCTAGCAAGT-3’
	5’-TCTTTTTTTTTTTTTTTTGAAGACATGTCGACGGCCATT-3’
	5’-TCAAAAAAAAAAAAAAAAGACTTTTTCTCCTCTCCAGCCCT-3’
